# Study of analgesic effect of earthworm extract

**DOI:** 10.1042/BSR20171554

**Published:** 2018-01-25

**Authors:** Wei Luo, Zhen-han Deng, Rui Li, Guo Cheng, Ronak Naveenchandra Kotian, Yu-sheng Li, Wen-ping Li

**Affiliations:** 1 Department of Orthopaedics, Xiangya Hospital, Central South University, Changsha, Hunan, P.R. China; 2 Department of Animal Science, College of Animal Science and Technology, Hunan Agricultural University, Changsha, Hunan, P.R. China; 3 Department of Orthopaedic Surgery, Victoria Hospital, Bangalore Medical College and Research Institute, Bangalore, India

**Keywords:** analgesic activity, earthworm extraction, 5-HT, NE

## Abstract

Pain represents a major clinical problem and one which has exercised generations of healthcare professionals. Earthworms are used as a traditional Chinese medicine, and have been applied pharmacologically and clinically since a long time in China. However, the analgesic effects of earthworm extract (EE) are seldom studied. Hence, we evaluated the analgesic effects of EE in mice. The obtained data showed that EE increased pain threshold and exhibited peripheral but not central analgesic effects in mice; evidenced by increased inhibition ratio in acetic acid writhing test and formalin test, whereas only slight increase in inhibition ratio in hot plate test and tail immersion test. In addition, EE decreased serum norepinephrine (NE), 5-hydroxytryptamine (5-HT), and nitric oxide (NO) synthase (NOS) concentration, similar to other analgesic drugs like morphine and aspirin. In a nutshell, the obtained data have demonstrated that EE has peripheral analgesic properties and could be used as a promising analgesic drug.

## Introduction

Although great progress has been made in improving human health, pain still dwells as the most cumbersome ailment in today’s hectic society. For many people, pain is more or less a permanent feature of their lives and has a profound impact on their quality of life. Therefore, management of pain is imperative so that suffering is minimized as much as possible. Opioids and NSAIDs are the prevalent pain medications used to alleviate the symptoms [1]. Although these pain medications have excellent analgesic effects, the array of adverse effects cannot be completely avoided [2]. Thus, given the disadvantages of these pain medications, it is indispensable to develop some novel analgesic drug with the same efficacy and fewer or no adverse effects.

Earthworms (Dilong in Chinese) are famous for their medicinal properties in China, and their pharmacological and clinical applications have been well documented [1,3]. Earthworm has dense nutritional contents [3] and its extracts have been traditionally applied to treat certain diseases by indigenous people in China [3]. Recently, earthworms have been paid more attention as emerging evidence has shown that earthworm extract (EE) has many bioactive molecules and biological functions. For example, many compelling studies have shown that EE has anti-ulceral [4], antioxidative [1], hepatoprotective [5], antimicrobial, anticancer [6], and anti-inflammatory [7] activities. Chang et al. have demonstrated that EE plays a role in Schwann cell migration and nerve regeneration via activation of matrix-degrading proteolytic enzymes (PAs and MMP2/9) through ERK1/2 and p38 signaling pathway [8]. Moreover, EE has been used to treat some diseases such as stroke and cardiovascular diseases [9]. Although, there exists various biological functions of EE, the analgesic property is rarely reported. In the present study, we primarily investigated analgesic effects of EE and obtained results showed that it has peripheral analgesic effects but no central analgesic effects and these analgesic activities could be due to decreased norepinephrine (NE), 5-hydroxytryptamine (5-HT), and serum nitric oxide (NO) synthase (NOS) concentration in serum and brain in mice by using well-established methods to evaluate central and peripheral antinociceptive effects [10–13].

## Materials and methods

### Preparation of EE

Ohira ii earthworms were cultured at a breeding center. The live earthworms were kept in distilled water at room temperature for one night to remove the attached mud. The following day, earthworms were homogenized with PBS (0.02 M, pH 7.2), and then centrifuged (5000×***g***, 4°C, 10 min). The supernatant was ultrafiltered in sequence with 100, 50, and 30 kDa Millipore ultrafilter (5000×***g***, 4°C, 10 min). The final ultrafiltered solution was collected. The protein concentration of final ultrafiltered solution (2.56 mg/ml) was measured and then stored at −20°C for the following experiments. We used 50% EE (EE with equal volume saline dilution, protein concentration: 1.28 mg/ml) and 100% EE (EE without saline dilution, protein concentration: 2.56 mg/ml) concentrations in our experiment as we found that doses less than 50% did not produce any analgesic effect. Henceforth, we considered only two doses in our experiments.

### Experimental animals

The Kunming mice (20 ± 2 g) and Sprague–Dawley rats (250 ± 20 g) used in the present study were purchased from the animal Laboratory Animal Center of Central South University, China. They were housed in a friendly and environmentally controlled condition with proper room temperature (23°C) and humidity (60%) and a 12-h light/12-h dark cycle. Both mice and rats had access to standard rodent food and water *ad libitum*. All animal care, handling, and surgical techniques followed protocols approved by the Animal Care and Use Committee of Central South University.

### Peripheral analgesic activities of EE

To evaluate whether EE has peripheral analgesic activities, we performed acetic acid writhing test and formalin test [10,14].

### Acetic acid writhing test

Acetic acid-induced writhing test was performed as reported previously [15]. Accordingly, 40 Kunming mice were randomly assigned into four treatment groups. Control group (CG), which received gavage with normal saline (0.1 ml/10 g); positive group (PG), which received gavage with aspirin (0.1 mg/kg), a standard analgesic drug [16]; low dosage group (LG), which received gavage with 50% EE (0.1 ml/10 g, protein concentration: 1.28 mg/ml) and high dosage group (HG), which received gavage with 100% EE (protein concentration: 2.56 mg/ml). The experiment lasted for 7 days. Two hours after gavage on the seventh day, mice were injected intraperitoneally with 0.2 ml of 0.6% acetic acid, and the number of writhes of each mouse was counted starting from 5 min up to 15 min and expressed as percentage protection. We arbitrarily chose 7 days of treatment in the present study to justify the fact that EE is a natural extract and a compound, and hence its analgesic effects cannot be ascertained as quickly as compared with other pain medications. Additionally, we chose gavage in the present study and the efficacy of EE could not be determined in a short period of time.

### Formalin test

The formalin test was performed as previously reported [17]. Forty Kunming mice were randomly assigned into four treatment groups similar to the acetic acid-induced writhing test. The experiment lasted for 7 days, and 2 h after gavage on the seventh day, mice were injected subcutaneously with 20 μl of 1.8 mol/l formalin into the plantar surface of one hind paw. Using licking response time as pain index, observers recorded licking response time in seconds. Phases were defined as follows: phase (0–5 min) and phase (20–40 min).

### Central analgesic activities of EE

To evaluate if EE has central analgesic effects, we performed hot plate test and tail immersion test [15,18].

### Hot plate test

All Kunming mice were subjected to a pretest on hot plate maintained at 55 ± 0.1°C to premeasure the latency time (the time for which mouse remains on the hot plate without licking or flicking of hind limb or jumping) of each mouse. Mice which had a latency time from 5 to 20 s were sorted out. The selected 40 mice were divided into four treatment groups as conducted in the acetic acid-induced writhing test except for PG in which mice received gavage with bucinnazine (15 mg/kg), an analgesic drug [19]. The experiment lasted for 7 days. Two hours after treatment on the seventh day, mice were placed on the hot plate and the latency time (in seconds) was measured in seconds after 60- and 120-min treatment except for positive CG measured after 30-min drug treatment. The percentage analgesia was calculated as reported previously [20].

### Tail immersion test

To evaluate the analgesic effects of EE, we conducted tail immersion test in mice based on previous study [18]. Forty Kunming mice were randomly assigned into four treatment groups as conducted in the acetic hot plate test. The experiment lasted 7 days. Two hours after treatment on the seventh day, the lower 5 cm section of tail of each mouse was immersed in an electric-heated thermostatic water bath of which the temperature was set to 55°C. The time in seconds for tail withdrawal from the water was recorded as the reaction time after 60- and 120-min treatment except for positive CG measured after 30-min drug treatment. In order to prevent tissue damage, a cut-off time of 15 s was imposed for all animals.

### Opioid receptor-dependent test

To evaluate if the analgesic effect of EE depends on opioid receptor, we conducted the following experiments. Briefly, mice were randomly assigned into five treatment groups. CG in which mice were injected intraperitoneally with normal saline; EE group (EG) in which mice were injected intraperitoneally with EE; EE + naloxone group (ENG) in which mice were injected intraperitoneally naloxone (5 mg/kg), an antagonist of opioid receptor [21], after 30 min injecting intraperitoneally with EE; morphine group (MG) in which group mice were injected intraperitoneally with morphine (10 mg/kg), an analgesic drug [22]; morphine + naloxone group (MNG) in which mice were injected intraperitoneally with naloxone after 30 min injecting intraperitoneally with morphine. The experiment lasted for 7 days. On the seventh day, the pain threshold of mouse was evaluated by using hot plate test after treatment every 30 min up to 120 min.

### NE and 5-HT determination

We preferred rat to mice in this experiment as we could not collect enough serum to determine the levels of NE and 5-HT in a mouse. Accordingly, 40 Sprague–Dawley rats were randomly assigned into four treatment groups as conducted in the acetic acid-induced writhing test. The experiment lasted 7 days. After 2-h treatment at seventh day, rats were injected subcutaneously with 20 μl of 1.8 mol/l formalin into the plantar surface of one hind paw. After 40-min formalin treatment, all rats were killed for collecting serum and brain samples. NE and 5-HT were measured by ELISA according to manufacturer’s protocols.

### NOS determination

Forty Kunming mice were randomly assigned into four treatment groups as conducted in the acetic acid-induced writhing test except for PG in which mice were injected intraperitoneally with morphine. The experiment lasted 4 days. After 2-h morphine treatment at fourth day, mice were injected subcutaneously with 20 μl of 1.8 mol/l formalin into the plantar surface of one hind paw. After 2-h treatment, all mice were killed for collecting serum and brain samples. NOS was measured by ELISA according to the manufacturer’s protocols.

### Statistical analysis

The values were expressed as mean ± S.D. [23]. All statistical analyses were performed using the SPSS 16.0 software (Chicago, IL, U.S.A.). Data were analyzed by independent sample *t* test compared with CG. A value of *P<0.05 was considered statistically significant (*P<0.05, **P<0.01).

## Results

### Peripheral analgesic activities of EE

**Figure 1:**
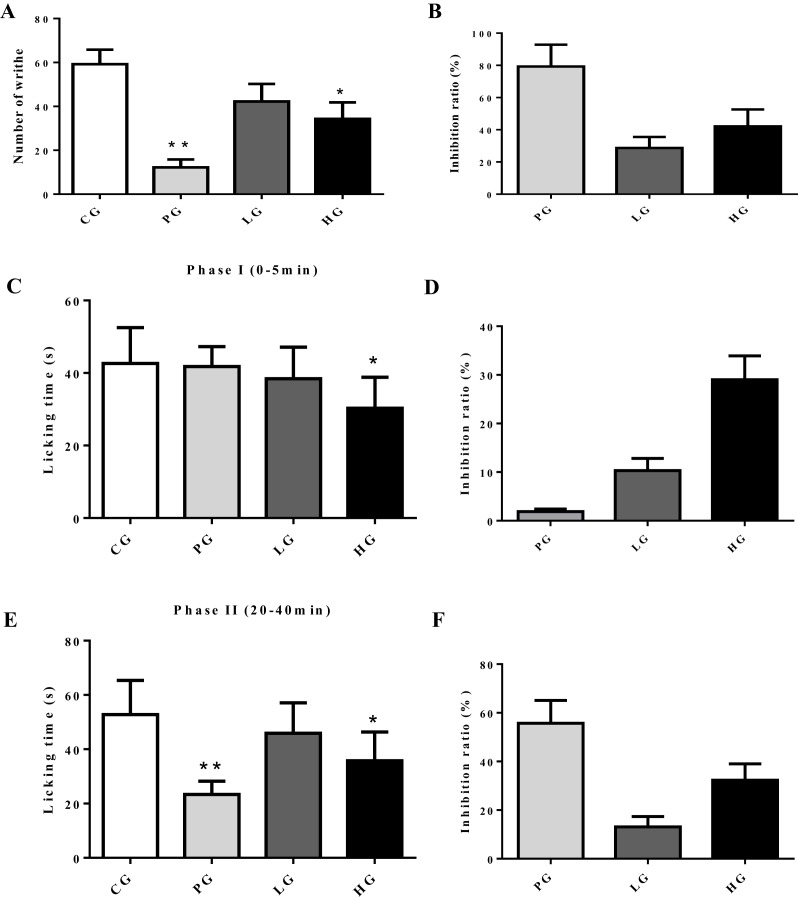
Peripheral analgesic activities of EE (**A,B**) The number of writhes of mice in acetic acid writhing test and its corresponding inhibition ratio. (**C**,**E**) The licking response time of mice in formalin test, and their corresponding inhibition (**D**,**F**). The data are presented as mean ± S.D., *n*=10. A value of *P<0.05 was considered statistically significant (*P<0.05, **P<0.01).

To evaluate whether or not analgesic activities of EE are through acting on peripheral tissues, we performed acetic acid writhing test and formalin test. As shown in Figure 1A,B, high dosage of EE significantly inhibited acetic acid-induced writhing response and increased pain threshold of mice evidenced by significant decrease in number of writhes and increased inhibition ratio compared with CG, while low dosage of EE exhibiting unremarked analgesic activity with soft decrease in number of writhes and increased inhibition ratio. Expectedly, positive drug aspirin showed significant analgesic activity with conspicuous decrease in number of writhes and increased inhibition ratio. These data suggest that EE may have certain peripheral analgesic activity. To further confirm this point, we conducted formalin test and the results showed that low dosage of EE exerted mild analgesic activity with mild reduction in licking time of Phase and Phase Ⅱ (Figure 1C,E) compared with CG, but high dosage of EE, like in acetic acid writhing test, showed significant decrease in licking time of Phase and Phase Ⅱ (Figure 1C,E) and corresponding increased inhibition ratio of Phase and Phase Ⅱ (Figure 1D,F), respectively. Aspirin plausibly decreased licking time of Phase Ⅱ (Figure 1C,E) and increased inhibition ratio of Phase Ⅱ (Figure 1F). Collectively, the analgesic activities of EE may be through acting on peripheral tissues in a dosage-dependent manner to alleviate pain.

### Central analgesic activity of EE

**Figure 2:**
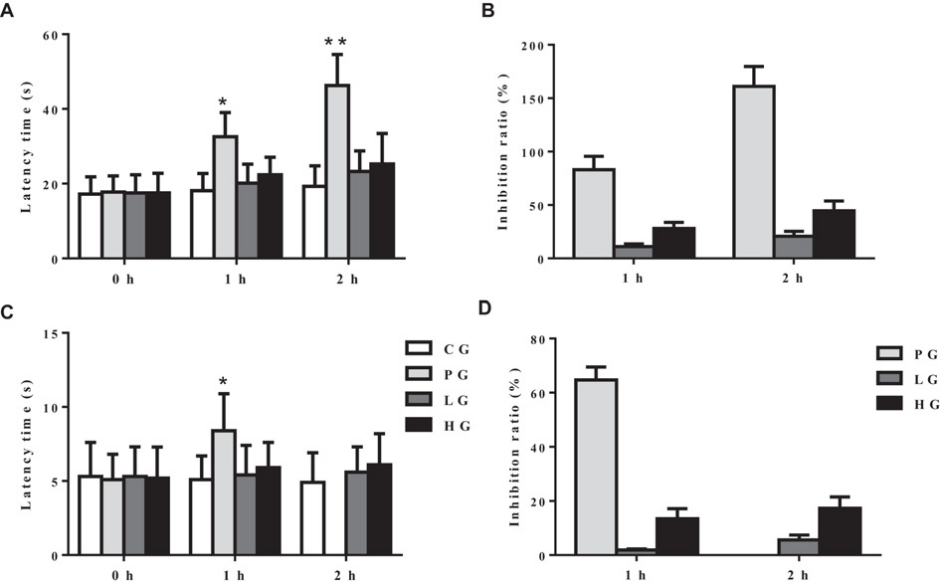
Central analgesic activities of EE (**A**,**B**) The latency time of mice in hot plate test and its corresponding inhibition ratio. (**C**,**D**) The reaction time of mice in tail immersion test and its corresponding inhibition. The data are presented as mean ± S.D., *n*=10. A value of *P<0.05 was considered statistically significant (*P<0.05, **P<0.01). The time in seconds for tail withdrawal in tail immersion test from the water was recorded as the reaction time.

Futhermore, to explore if analgesic effects of EE were associated with central nervous system (CNS), we performed hot plate test and tail immersion test. In hot plate test, positive analgesic drug bucinnazine significantly increased response latency both 1 and 2 h (Figure 2A,B) after treatment compared with CG. Surprisingly, in both low and high dosages of EE, there was no significant analgesic effect. But, a slight increase in response latency after both 1 and 2 h (Figure 2A,B) of EE treatment in HG was observed. Additionally, tail immersion test also showed similar results to both low and high dosages of EE that it failed to exert marked analgesic effects after both 1 and 2 h (Figure 2C,D) of EE treatment in HG group. In a nutshell, the obtained data suggested that EE has no notable central analgesic effects.

### Analgesic effect of EE independent of opioid receptor

**Figure 3:**
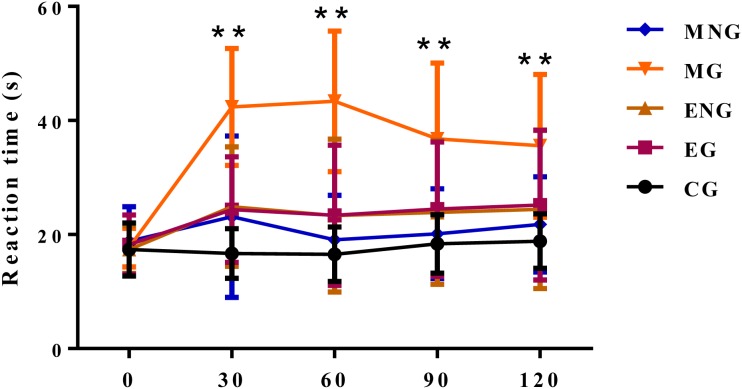
Analgesic activity of EE independent of opioid receptor The data are presented as mean ± S.D., *n*=10. A value of *P<0.05 was considered statistically significant (*P<0.05, **P<0.01).

Many compelling studies have shown that opioid receptor is responsible for analgesic effects and is a target for many analgesic drugs. In the present study, we used morphine, a classical analgesic drug which is an agonist of opioid receptor [24] and naloxone, an antagonist of opioid receptor [21], to study if the analgesic effect of EE is dependent on opioid receptor. As shown in Figure 3, compared with CG, treatment with EE slightly increased response latency at every time point (30, 60, 90, and 120 min) in mice, but intriguingly, these mild analgesic effects failed to be alleviated by naloxone. On the contrary, treatment with naloxone significantly blocked morphine-mediated analgesic effects evidenced by decreased response latency in mice. These results suggested that analgesic effects of EE are independent of opioid receptor.

### NE, 5-HT, and NOS concentration in serum and brain

**Figure 4:**
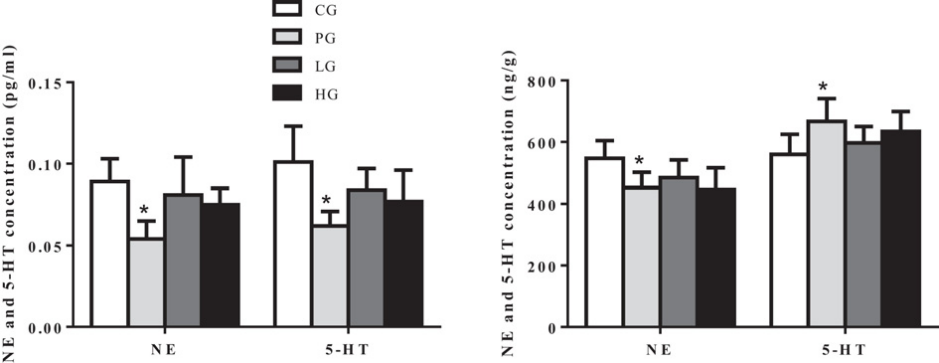
Effects of EE on NE and 5-HT concentration in serum and brain of rats (**A**) NE and 5-HT concentration in serum. (**B**) NE and 5-HT concentration in brain. The data are presented as mean ± S.D., *n*=10. A value of *P<0.05 was considered statistically significant (*P<0.05).

**Figure 5:**
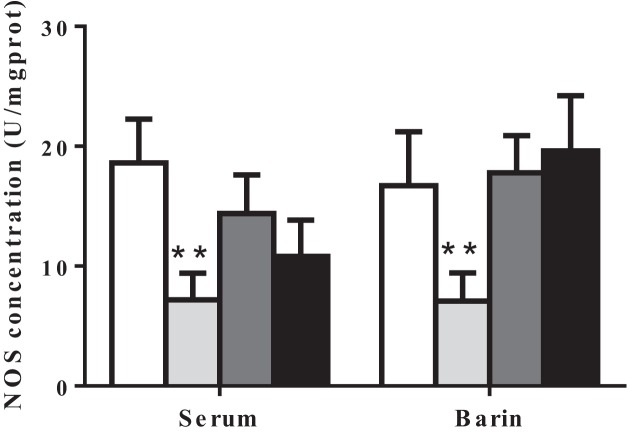
Effects of EE on NOS concentration in serum and brain of mice The data are presented as mean ± S.D., *n*=10. A value of *P<0.05 was considered statistically significant (**P<0.01).

To further confirm the analgesic effects of EE, we measured NE, 5-HT, and NOS levels in serum and brain because of their known role as antinociceptives [25,26]. As shown in Figure 4, both high and low doses of EE tended to lower serum NE and 5-HT concentration, especially high dosage of EE significantly reduced serum 5-HE concentration, but the analgesic effects of EE were not as good as positive drug aspirin (Figure 4A). Intriguingly, the NE and 5-HT levels in brain exhibited opposite results of which both high and low dosage of EE decreased NE concentration, while on the contrary, increased 5-HT concentration in brain (Figure 4B). Positive analgesic drug aspirin also exhibited similar pattern, significantly decreased NE concentration but increased 5-HT concentration in brain (Figure 4B). In addition, EE, both high and low dosages, tended to decrease serum NOS concentration and high dosage of EE reached up statistical difference, but in brain, EE exhibited no effects on NOS concentration (Figure 5). As expected, positive drug morphine significantly decreased NOS concentration in serum and brain (Figure 5). The obtained data suggested that analgesic effect of EE is due to altering the levels of NE, 5-HT, and NOS concentration in serum and brain.

## Discussion

Many studies have uncovered the neuronal mechanisms of pain and have shown that both peripheral and central nociceptive systems contribute to pain during inflammation and nerve injury [27]. To alleviate the pain symptoms, medications such as morphine, diclofenac sodium, and cannabinoids have been developed according to their action target [28,29]. Although, these drugs have been extensively used in clinic, unfortunately these come with inevitable insults to individual’s health. In the present study, we have found that EE increased pain threshold and exhibited peripheral but not central analgesic effects in mice. Although, we could not demonstrate any toxic effects of EE in our experiments, to the best of our knowledge, it exhibited no gross toxic effects. Furthermore, our observation is in tune with the recent studies conducted by Chang et al. [30] and Li et al. [31], who have shown that EE can improve peripheral nerve regeneration and exhibits analgesic activity. Simultaneously, we used naloxone, an antagonist of opioid receptor [21] which plays a pivotal role in central nociceptive system, to block opioid receptor, and found that naloxone exhibited no effects on analgesic effects of EE in mice.

In addition, many compelling studies have shown that neural transmitters such as 5-HT, NE, and NO are involved in analgesia in both peripheral and central nociceptive system [25,26,32]. Some researchers have found that increased peripheral 5-HT and NE can exaggerate pain [25,33]. Ashina et al. [26] have shown that inhibition of NOS had an analgesic effect in chronic tension-type headache. Thus, in the present study, we also measured the levels of these neural transmitters in serum and brain, and found that EE decreased serum 5-HT, NE, and NOS concentration, as shown by positive analgesic drugs (aspirin and morphine). The decreased serum 5-HT, NE, and NOS concentration provided an evidence for the peripheral analgesic effects of EE because decreased neural transmitters can reduce the sensory receptor’s activation in primary afferent fiber and sensitivity of nociceptor in peripheral tissues and thus exerting its peripheral analgesic effects [25,32]. Moreover, NOS is a key enzyme for NO synthesis from arginine, and NO is an important transmitter in pain pathways in both peripheral and central nociceptive system [26,34]. EE-mediated decreased serum NOS implies decreased NO production, which further contributes to antinociceptive effects [35]. Furthermore, pain generation is closely associated with inflammatory response [36] and some authors have demonstrated that EE has both anti-inflammatory and anti-oxidative properties [4,31]. As a result, it is yet to be confirmed if the anti-inflammatory property of EE indeed contributes to its analgesic effect. Recently, a study from Li et al. [31] has supported this speculation, in which they have purified and characterized two novel peptides, VQ-5 and AQ-5, from the coelomic fluid of the earthworm which exhibits analgesic activity and anti-inflammatory effects through inhibiting mitogen-activated protein kinase signaling pathway. Also, one must note that EE used in the present study is a compound and it may exist with other active bioactive molecule(s) besides VQ-5 and AQ-5 that exert analgesic effects. Additionally, we have conducted series of experiments (data not shown) and have purified two components of EE (molecular weights: 21.6 and 28.2 kDa, respectively) which may yield better results in our future studies.

Our study has several limitations. First, we could not hypothesize the mechanism responsible for our observations in the present study due to lack of knowledge of the active molecule in EE. Since our primary objective in the present study was to analyze if EE had any analgesic effect at all, we would like to further our research in that direction in the future. Second, we could not demonstrate any toxic effects of EE in our experiment, but to the best of our knowledge, it exhibited no gross toxic effects. However, the possibility of adverse effects cannot be ignored. Henceforth, future research should focus on isolating the active entity that activates the cascade of pain inhibition and also study the adverse effects which are currently unknown.

In conclusion, we have found that EE has peripheral analgesic effects. Additionally, EE can decrease serum NE, 5-HT, and NOS concentration in mice which may have contributed to the analgesic effects. This is the first study to report the analgesic effects of EE.

## Abbreviations

CG: control group
EE: earthworm extract
ERK 1/2: extracellular signal-regulated kinase 1/2
HG: high dosage group
5-HT: 5-hydroxytryptamine
LG: low dosage group
MMP 2/9: matrix metalloproteinase 2/9
NSAID: nonsteroidal anti-inflammatory drug
NE: norepinephrine
NOS: nitric oxide synthase
PA: plasminogen activator
PG: positive group
